# IL-4 driven transcription factor FoxQ1 is expressed by monocytes in atopic dermatitis and stimulates monocyte migration

**DOI:** 10.1038/s41598-017-17307-z

**Published:** 2017-12-04

**Authors:** Ilja Ovsiy, Vladimir Riabov, Ioannis Manousaridis, Julia Michel, Kondaiah Moganti, Shuiping Yin, Tengfei Liu, Carsten Sticht, Elisabeth Kremmer, Martin C. Harmsen, Sergij Goerdt, Alexei Gratchev, Julia Kzhyshkowska

**Affiliations:** 1 Department of Dermatology, Venerology and Allergology, Medical Faculty Mannheim, University of Heidelberg, Mannheim, Germany; 2 Institute for Transfusion Medicine and Immunology, Medical Faculty Mannheim, University of Heidelberg, Mannheim, Germany; 3 Center for Medical Research, Medical Faculty Mannheim, University of Heidelberg, Mannheim, Germany; 4 Institute of Molecular Immunology, Helmholtz Zentrum München, German Research Center for Environmental Health (GmbH), Munich, Germany; 5 Department of Pathology and Laboratory Medicine, University of Groningen, University of Groningen Medical Center, Groningen, The Netherlands; 6 Institute of Carcinogeneis, NN Blokhin Russian Cancer Research Center, Moscow, Russia; 7 Red Cross Blood Service Baden-Württemberg–Hessen, Friedrich-Ebert Str. 107, Mannheim, Germany

## Abstract

Monocytes are actively recruited at sites of chronic inflammation. However, molecular factors involved in this process are not fully elucidated. Here, we show that cytokine IL-4 which is implicated in the development of chronic inflammatory disease atopic dermatitis (AD) induces expression of transcription factor FoxQ1 in human monocytes and macrophages. FoxQ1 mRNA levels were elevated in monocytes of AD patients compared to healthy donors. Overexpression of FoxQ1 in RAW 264.7 monocytic cells facilitated their migration towards MCP-1 and was associated with decreased expression of migration-regulating genes (claudin 11 and plexin C1). Furthermore, FoxQ1 overexpression in RAW cells accelerated TNFα secretion after LPS challenge. Overall, our results indicate that FoxQ1 stimulates monocyte motility, increases pro-inflammatory potential, and directs monocyte migration towards MCP-1 that is crucial for monocyte influx into inflammatory sites. This mechanism could contribute to the pathogenesis of chronic inflammatory disorders such as AD.

## Introduction

Monocytes are critical components of inflammatory reactions. Increased numbers of infiltrating monocytes is characteristic for acute and chronic inflammatory disorders including atherosclerosis, chronic liver diseases, and atopic dermatitis (AD)^1–3^. Damaged and inflamed tissue attracts and recruits circulating monocytes by secreting MCP-1. This key monocyte recruiting chemokine is secreted by various cell types including epithelial cells, fibroblasts, endothelial cells and monocytes^3,4^. At the site of inflammation, monocytes differentiate into mature macrophages under control of local tissue-derived and inflammatory factors. In case of an acute inflammatory response to pathogens or trauma, monocyte-derived macrophages exert their anti-bacterial function, clear the infection, and stimulate the resolution of inflammation and healing. The inability of macrophages to control the resolution phase results in development of chronic inflammation. The balance of cytokines and chemokines produced by macrophages, T-cells and other immune cells is critical for efficient resolution of inflammation. Key prototype cytokines involved in the regulation of acute inflammation and its resolution are IFN-γ and IL-4. IFN-γ induces acute phase of inflammation and is recognized as prototypic M1-polarizing factor^5^. IL-4 antagonizes effects of IFN-γ and stimulates alternative macrophage activation which is associated with extracellular matrix remodeling, repair and resolution of inflammation^5,6^. However, increased systemic and local IL-4 levels are also found in chronic Th2-associated inflammatory conditions such as asthma and AD^7–10^. AD is a chronic inflammatory skin disease which is characterized by abnormalities in skin barrier function (itchy, red, swollen skin), and immune dysregulation^10,11^. In AD immune dysregulation involves increased infiltration of IL-4-producing Th2 cells, eosinophils and macrophages expressing elevated levels of the scavenger receptor CD163 and mannose receptor (CD206)^2,12,13^. Recently, forkhead box transcription factor FoxQ1 has been identified as one of the hubs in IL-4 activated transcriptional networks in human macrophages^14^. However, despite involvement of FoxQ1 in migration, invasion and proliferation of tumor cells, its functional role in monocytes/macrophages and association with human pathologies other than cancer remained unknown^15–18^.

In this study we demonstrate that FoxQ1 expression is upregulated in circulating monocytes of patients with AD. Utilizing *ex vivo* cultured monocyte-derived macrophages from healthy donors we revealed stringent regulation of FoxQ1 expression by Th1/Th2-associated cytokines. Using gain-of function approach we demonstrated that FoxQ1 markedly enhances migration of monocytes towards chemokine MCP-1. By analyzing FoxQ1-regulated genes we revealed that both activation of cytoskeleton dynamics and suppression of negative regulator of migration Plexin C1 are associated with FoxQ1-inducible migration. Overall, our results indicate that FoxQ1 is critical factor for IL-4-mediated recruitment of monocytes in chronic inflammatory conditions.

## Results

### FoxQ1 expression in macrophages is stimulated by IL-4 and suppressed by IFN-γ

**Figure 1:**
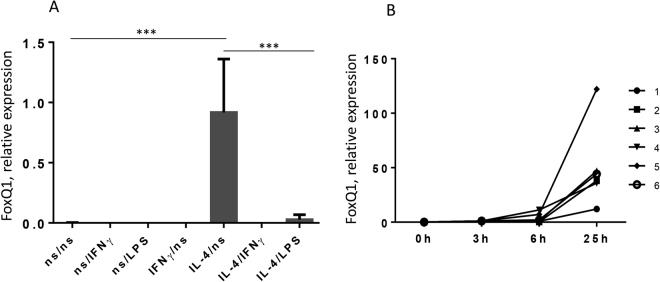
The analysis of FoxQ1 mRNA expression in primary human macrophages. (**A**) Macrophages of 8 healthy donors were stimulated with IL-4 or left non-stimulated (ns) for 6 days and then re-stimulated with IFN-γ or LPS for 6 hours. FoxQ1 expression was assessed using RT-qPCR. The data are mean ± SD. ***p < 0.001, one-way ANOVA with Tukey’s multiple comparison test. (**B**) The dynamics of FoxQ1 mRNA expression was studied in IL-4-stimulated monocytes isolated from 6 healthy donors using RT-qPCR.

Recently, the Th2-associated cytokine IL-4 was shown to enhance expression of FoxQ1 in human monocyte-derived macrophages^14^. However, the regulation of its expression by Th1/Th2 cytokine balance was not studied. It was previously found in our laboratory that macrophages induced by IL-4 are plastic cells, and can be reprogrammed in culture conditions^19^. Therefore, the regulation of FoxQ1 expression was analyzed in macrophages upon their reprogramming by cytokines and bacterial products. Monocytes isolated from buffy coats were cultured without stimulation or in the presence of IL-4 or IFN-γ. On the sixth day of culture the cells were restimulated with IFN-γ or LPS for 6 h and FoxQ1 expression was analysed using RT-qPCR. Indeed, IL-4 stimulation for 6 days significantly increased FoxQ1 expression in macrophages (p < 0.001) whereas its expression was negligible in non-stimulated and IFN-γ-stimulated macrophages (Fig. 1A). Re-stimulation of IL-4-treated macrophages with IFN-γ or LPS resulted in rapid downregulation of FoxQ1 mRNA expression indicating stringent regulation by Th1/Th2 cytokines and bacterial products (Fig. 1A). We further examined the dynamics of FoxQ1 expression in response to IL-4 stimulation using monocytes from 5 healthy donors. Increased expression of FoxQ1 gene was evident already 3 h after addition of IL-4 and rapidly increased between 6 h and 25 h of stimulation indicating direct and tight regulation of FoxQ1 expression by IL-4 (Fig. 1B and Supplementary Fig. 1).

**Figure 2:**
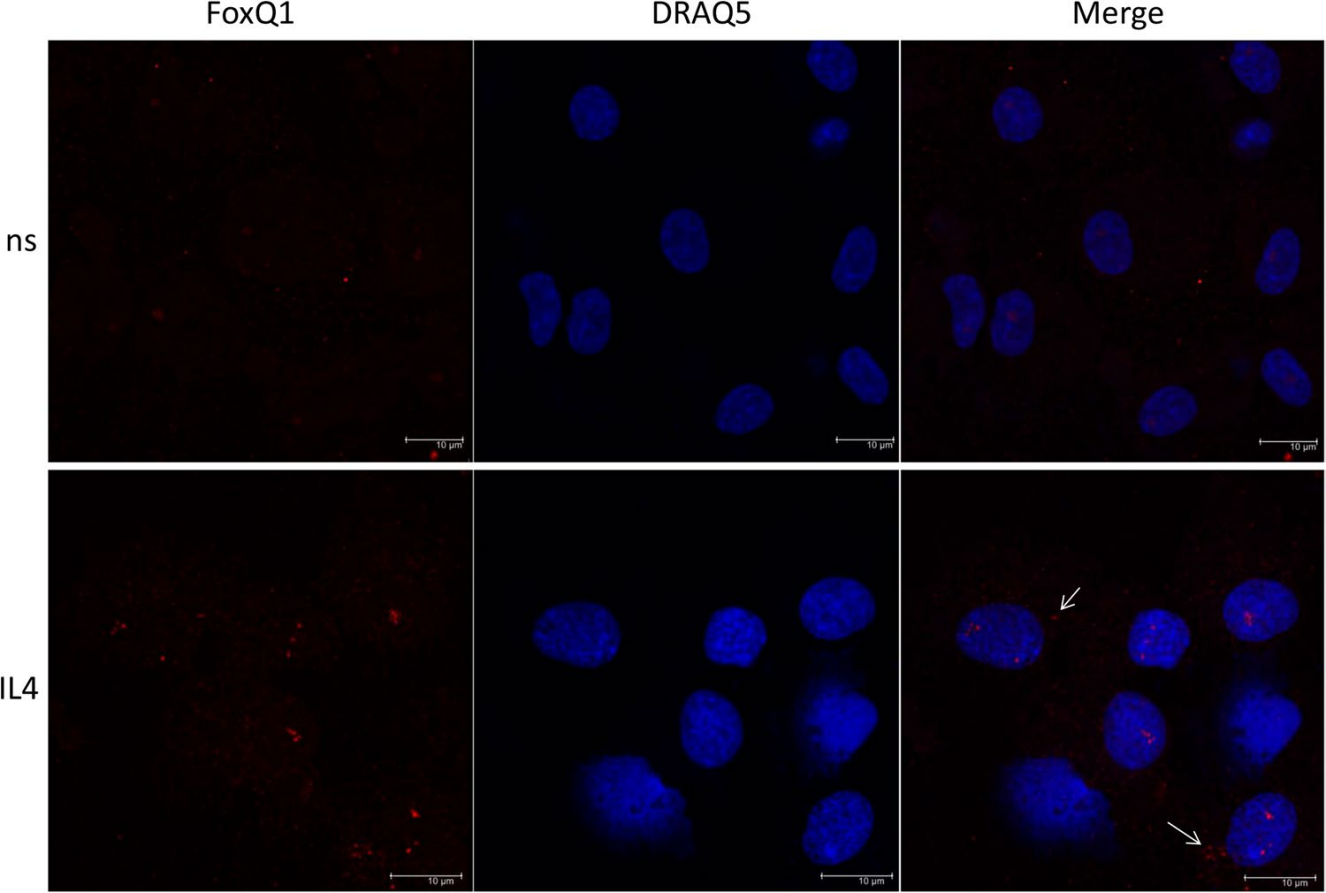
The analysis of FoxQ1 protein expression in primary human macrophages. Monocytes were isolated from blood of healthy donors (n = 3) and cultured for 6 days without stimulation (ns) or in the presence of IL-4. FoxQ1 expression (red) was assessed by immunofluorescent staining/confocal microscopy using goat anti-human FoxQ1 abs. Nuclei were visualized using DRAQ5 (blue). White arrows indicate cytoplasmic presence of FoxQ1 in macrophages. Representative images are shown. Scale bars: 10 µm.

On the protein level FoxQ1 was weakly expressed in non-stimulated macrophages (ns) and more pronouncedly in IL-4 stimulated monocyte-derived macrophages as detected by immunofluorescent staining/confocal microscopy on day 6 of culture (Fig. 2). The pattern of protein expression revealed preferential nuclear localization of FoxQ1 in human primary macrophages (Fig. 2). However, in contrast to human colorectal cancer cell line WIDR that was used as a positive control and showed nearly exclusive nuclear localization of FoxQ1, in macrophages its expression was also detectable in the cytoplasm (Fig. 2 and Supplementary Fig. 2)

### FoxQ1 expression is enhanced in monocytes of AD patients

**Figure 3:**
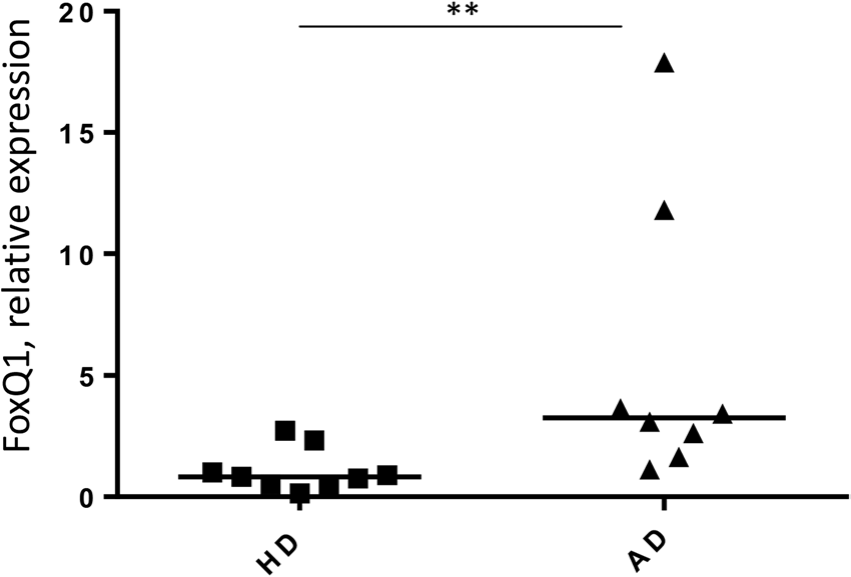
RT-qPCR analysis of FoxQ1 mRNA expression in patients with atopic dermatitis. Monocytes were isolated from blood of patients with AD (n = 8) and healthy donors (n = 9) and directly used for RNA isolation. **p = 0.0016, Mann-Whitney U test.

Increased IL-4 expression is found during chronic inflammatory conditions (e.g. asthma, allergic inflammation and AD) that are also characterized by increased influx of monocytes/macrophages to the lesions^8,20,21^. Since IL-4 plays an important role in the pathogenesis of atopic dermatitis (AD), we further tested whether expression of FoxQ1 is changed in blood monocytes from AD patients compared to healthy donors. The analysis revealed significant increase in expression of FoxQ1 in monocytes of AD patients compared to healthy donors (p = 0.0016) suggesting potential implication of FoxQ1 in pathogenesis of AD (Fig. 3).

### FoxQ1 expression accelerates TNFα production but does not affect scavenging activities of RAW 264.7 cells

To study functional role of FoxQ1 in monocytes we generated murine monocyte/macrophage RAW 264.7 clones stably expressing FoxQ1 (Supplementary Fig. 3). This monocyte cell line was selected due to negligible levels of endogenous FoxQ1 expression (data not shown). First, we have studied whether basic macrophage activities (inflammatory response, phagocytosis, and endocytosis) are affected by FoxQ1 overexpression.

**Figure 4:**
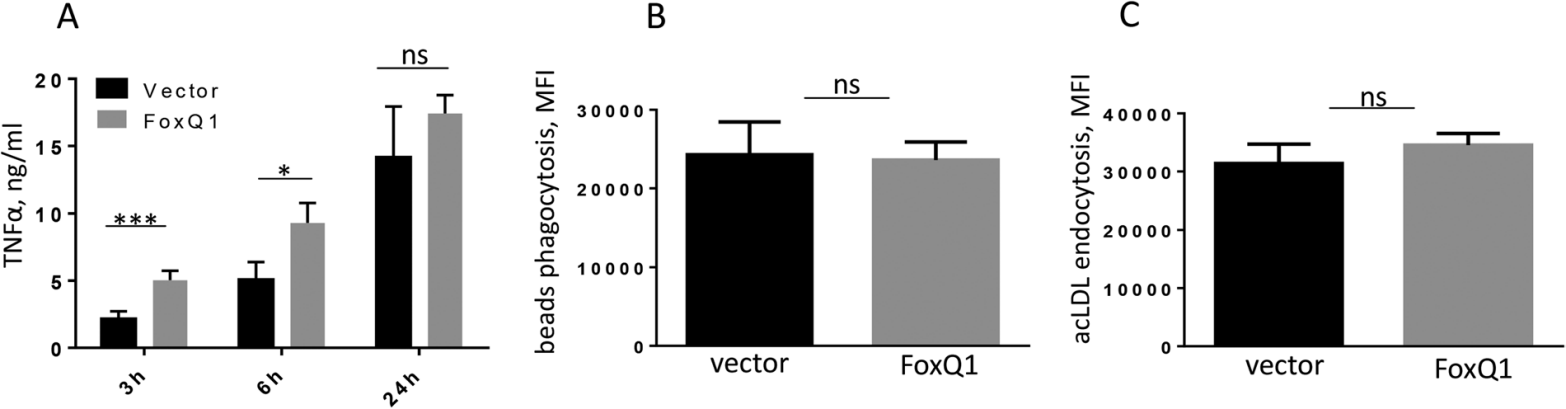
The analysis of inflammatory response and scavenging activity in FoxQ1 expressing RAW 264.7 cells. (**A**) FoxQ1 and empty vector-transfected cells were stimulated with LPS for 3 h, 6 h and 24 h, and TNFα production was analyzed by ELISA. The data are mean ± SD for two vector and two FoxQ1 clones analyzed in triplicates. *p < 0.05, ***p < 0.001; One-way ANOVA with Sidak’s multiple comparison test. (**B**) Phagocytosis of fluorescent latex beads in FoxQ1 and vector clones was performed for 3 h and assessed using flow cytometry. The data are mean fluorescence intensity (MFI) ± SD for 3 independent experiments with 3 FoxQ1 and 3 empty vector clones, ns - not significant, two-tailed Student’s t test. (**C**) Endocytosis of acLDL-Alexa488 was performed for 30 min and measured using flow cytometry. The data are MFI ± SD for 3 independent experiments with 3 FoxQ1 and 3 empty vector clones, ns - not significant, two-tailed Student’s t test.

To assess inflammatory response, RAW-FoxQ1 cells and RAW-vector cells were stimulated by LPS, and production of TNFα was quantified by ELISA 3 h, 6 h and 24 h after LPS challenge. Presence of FoxQ1 resulted in significant acceleration of TNFα secretion 3 h (p < 0.001) and 6 h (p < 0.05) after LPS stimulation indicating that FoxQ1 increases pro-inflammatory potential of monocytes/macrophages (Fig. 4A). TNFα production in unstimulated RAW-vector and RAW-FoxQ1 cells was below detection levels. In contrast, macrophage scavenging activity was not affected by FoxQ1 (Fig. 4B and C). Specifically, flow cytometry analysis of phagocytosis of fluorescent latex beads demonstrated that the presence of FoxQ1 had no effect on the phagocytic uptake in macrophages (Fig. 4B). Endocytosis is another essential macrophage function related to the resolution of inflammation and tissue remodeling^22^. Thus, we have compared endocytic uptake of fluorescently labeled acetylated low density lipoprotein (acLDL, common ligand of multiple scavenger receptors) by RAW-FoxQ1 and RAW-vector clones, and found that both cell types showed similar rates of acLDL uptake (Fig. 4C).

### FoxQ1 induces migration of RAW 264.7 cells towards MCP-1

**Figure 5:**
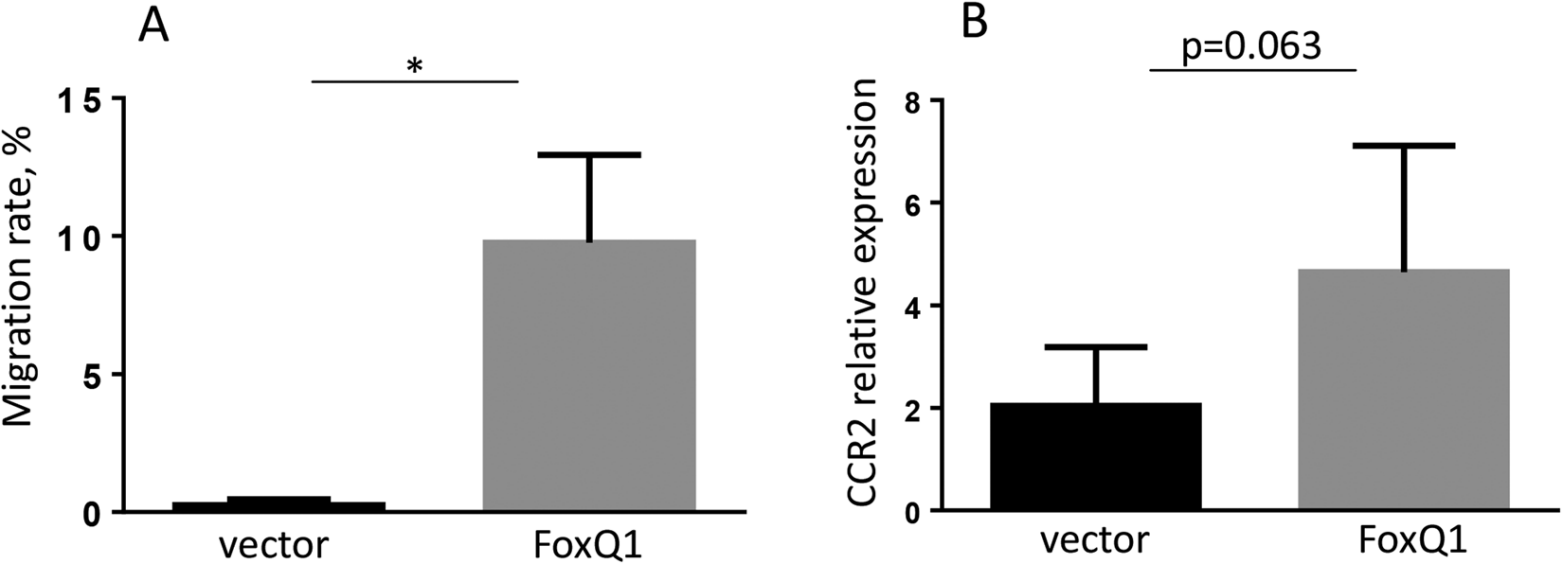
The analysis of RAW 264.7-FoxQ1 migration towards MCP-1. **(A**) Migration of RAW 264.7 cells towards MCP-1 (100 ng/ml) was studied using Neuro Probe chemotaxis chamber. The data are combined for 5 FoxQ1 and 5 vector-transfected clones and presented as mean ± SD. *p < 0.05, Kruskal-Wallis test with Dunn’s post-hoc test. (**B**) CCR2 expression was analyzed by RT-qPCR in 4 vector-transfected vs 5 FoxQ1 RAW 264.7 clones. The data are presented as mean ± SD, p = 0.063, Mann-Whitney U test.

Since FoxQ1 expression was found to be elevated in the circulating monocytes of patients with AD, the disease associated with accumulation of macrophages in skin lesions, we assessed another essential function of circulating monocytes – migration. The ability of FoxQ1 to stimulate migration towards key inflammatory chemotactic cytokine MCP-1 was investigated. MCP-1 is produced by keratinocytes, fibroblasts, macrophages, and other immune cells in sites of tissue inflammation^3,4^, and instructs circulating monocytes to enter the lesion. In our study FoxQ1 significantly increased migration of RAW cells towards MCP-1 (p < 0.05) (Fig. 5A). Notably, all FoxQ1 clones uniformly increased their migratory ability towards MCP-1 (Fig. 5A).

Since MCP-1 had pronounced effect on migration of FoxQ1 expressing RAW cells, we tested whether FoxQ1 stimulates expression of MCP-1 receptor CCR2, and found that there was a tendency for increased expression of CCR2 in RAW-FoxQ1 clones that did not reach statistical significance (Fig. 5B, p = 0.063, Mann-Whitney U test).

Our data indicated that FoxQ1 is crucial factor inducing migration of monocytes towards MCP-1. This finding also correlates with the fact, that recruitment of circulating monocytes is significantly increased in patients with AD^2,21^.

### Identification of FoxQ1 inducible genes involved in monocyte migration

**Figure 6:**
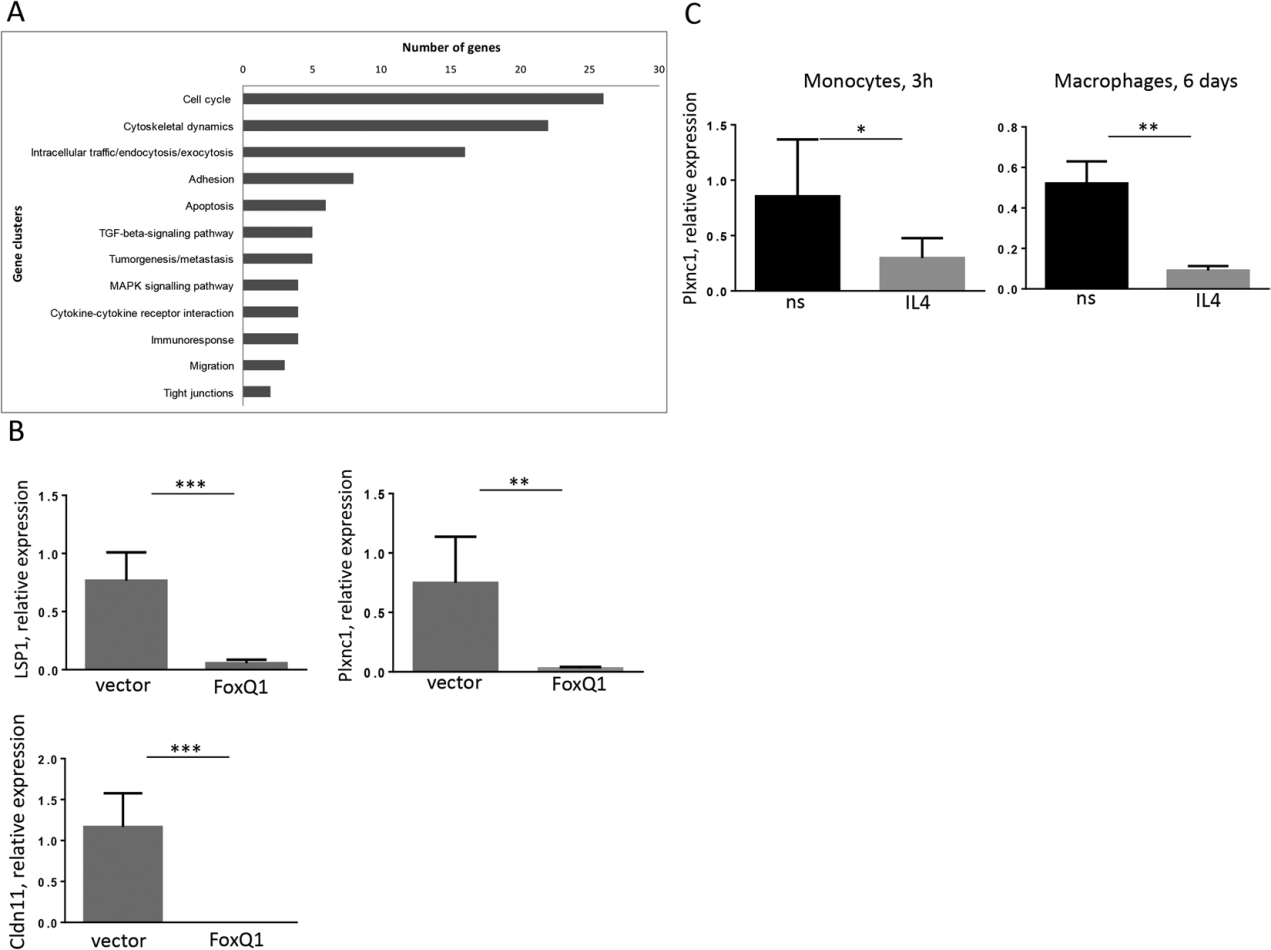
FoxQ1 target genes are involved in monocyte migration. (**A**) RAW 264.7 clones stably expressing mouse FoxQ1 and empty vector clones were subjected to Affymetrix microarray analysis and clusters of differentially expressed genes related to multiple cellular functions were identified. (**B**) Differentially expressed genes responsible for cell migration (LSP1, Cldn11, Plxnc1) were verified in RAW 264.7 FoxQ1 clones using RT-qPCR. The data are mean ± SD for 5 FoxQ1 and 4 empty vector clones. **p < 0.01, ***p < 0.001, two-tailed Student’s t-test. (**C**) The expression of plexin C1 was measured in monocytes of healthy donors (n = 4) stimulated with IL-4 for 3 h and in monocyte-derived macrophages (n = 4) stimulated with IL-4 for 6 days using RT-qPCR. The data are mean ± SD, *p < 0.05, **p < 0.01, two-tailed Student’s t-test.

In order to identify FoxQ1-regulated genes that are involved in monocyte/macrophage migration, we compared five RAW 264.7 clones expressing FoxQ1 and four empty vector clones using Affymetrix microarrays. The analysis of microarrays data revealed several clusters of differentially expressed genes involved in various biological processes including cytoskeletal dynamics, migration and formation of tight junctions (Fig. 6A). Among them 21 genes responsible for cytoskeletal remodeling were upregulated in FoxQ1 expressing clones (Supplementary Table 1). In contrast, a cluster of 15 genes involved in the modulation of cellular migration and metastasis was downregulated in FoxQ1 expressing clones (Supplementary Table 2). Validation of microarray data using RT-qPCR confirmed downregulation of the expression of Lsp1 (lymphocyte specific 1), Plxnc1 (plexin C1) and Cldn11 (claudin 11) genes known to play a role in cell migration^23–26^ (Fig. 6B). Next, we have examined whether expression of these genes is inhibited in human monocytes and macrophages under IL-4 stimulation. Lsp1 expression was not decreased in monocytes/macrophages stimulated with IL-4 for 3 h or 6 days suggesting regulation by other factors. The expression of claudin 11 was not detected in human primary monocytes and monocyte-derived macrophages. Of note, significant inhibition of plexin C1 expression was found in human monocytes stimulated with IL-4 for 3 h (p < 0.05) and in monocyte-derived macrophages differentiated with IL-4 for 6 days (p < 0.01) suggesting that Plexin C1 repression is associated with increased FoxQ1 expression (Fig. 6C). However, there was no direct correlation between levels of FoxQ1 and Plexin C1 expression in the individual donors (data not shown) suggesting that the magnitude of cellular response to FoxQ1 can be defined by the additional unknown factors, which expression or activity differs between macrophages from individual donors.

## Discussion

The ability of macrophages to be rapidly re-polarized by changing cytokine milieu (i.e. macrophage plasticity) is widely reported^19,27–29^. On the molecular level re-polarization of macrophages with prototypic Th1/Th2 cytokines IFN-γ and IL-4 involves dynamic changes in expression of polarization markers such as CD206, arginase I, iNOS, chitinase-like proteins and others^30–32^. The results of our study extend the list of macrophage polarization markers and propose FoxQ1 as IL-4-responder gene that is tightly regulated by Th1/Th2 cytokine balance. In addition, FoxQ1 can serve as a potential biomarker of type 2 inflammation as discussed in our previous review^33^.

The accumulation of macrophages in skin lesions of AD patients is a known fact^2,21^. It was demonstrated that macrophage accumulation in skin lesions is increased with disease severity^21^. However, mechanisms related to monocyte recruitment in AD are insufficiently studied. Our study demonstrates that IL-4-driven factor FoxQ1 is a crucial transcription factor responsible for increased monocyte migration. Thus, FoxQ1 is a candidate transcription factor that can be responsible for increased monocyte influx in IL-4-related pathologies such as AD. Indeed, we demonstrated that IL-4 induced expression of FoxQ1 in monocytes and monocyte-derived macrophages. Moreover, its expression was significantly increased in monocytes of AD patients compared to healthy controls. Previous studies demonstrated that IL-4 was capable of inducing the expression of monocyte chemoattractant MCP-1 in keratinocytes and its levels were elevated in AD patients^21,34^. Using RAW 264.7 monocyte/macrophage cell line we showed that overexpression of FoxQ1 resulted in significant activation of monocyte migration towards MCP-1 gradient. On the gene expression level this was associated with downregulation of genes responsible for the control of cell migration (claudin 11 and plexin C1) as well as increase in expression of chemokine receptor CCR2. Thus, our data add on to previously published studies and suggest that IL-4 can induce migration of monocytes towards sites of chronic inflammation by simultaneous upregulation of FoxQ1 in circulating monocytes and MCP-1 locally at the site of inflammation. Interestingly, Vogel *et al*. showed that IL-4 stimulated macrophages were characterized by enhanced velocity and migrated towards MCP-1 and other chemoattractants over longer distances and in a more organized fashion compared to non-stimulated and IFN-γ stimulated cells. Moreover, this migration pattern was associated with difference in cytoskeletal rearrangements in different subtypes of macrophages^35^. However, molecular factors responsible for increased macrophage motility in IL-4 stimulated cells were not examined. The results of our study suggest that acquisition of migratory phenotype in monocytes/macrophages is at least partially associated with IL-4-dependent induction of transcription factor FoxQ1 that affects expression of genes involved in cytoskeletal remodeling. Among them plexin C1 is responsible for inhibition of monocyte migration and is rapidly downregulated in IL-4 stimulated macrophages^24^. In addition, we have demonstrated that besides significant impact of FoxQ1 expression on monocyte migration it also accelerated TNFα production in response to pro-inflammatory stimuli which can potentially contribute to development of inflammatory disorders. Of note, it was previously reported that TNFα levels are increased in patients with AD and targeting this cytokine is one of therapeutic strategies in this disease^36–38^. TNFα is known to induce recruitment of cell types involved in pathogenesis of AD including eosinophils and CD4+ T cells^39–43^. In addition, TNFα induces activation of endothelial cells that facilitates adhesion and transmigration of multiple cell types in the area of inflammation^44–47^. Interestingly, in a recent study hepatocellular carcinoma cells expressing FoxQ1 were shown to increase recruitment of macrophages through CCL2 production. Macrophage depletion in FoxQ1 expressing tumors resulted in significant decrease in TNFα expression^48^. This data partially corresponded to our results. However, study by Xia *et al*. focused on FoxQ1 expression in tumor cells and did not take into consideration eventual expression of FoxQ1 in tumor macrophages. Overall, our data identify FoxQ1 as a novel IL-4-induced transcription factor in macrophages that facilitates their migration in chronic inflammatory conditions.

## Materials and Methods

### Atopic dermatitis patients

Eight patients (18–60 years old) with clinically proven atopic dermatitis and high serum IgE levels were involved in the study. All biological material has been collected, processed and handled in accordance with the German legislation on bioethics (approval by the Mannheim Ethics Committee of the Medical Faculty Mannheim, University of Heidelberg, approval number 112/05) and informed consent was obtained from all patients.

### Primary macrophages and cell lines

Human peripheral blood mononuclear cells were isolated and cultivated as described^49^. Briefly, the cells were purified from individual buffy coats by sequential density gradient centrifugations followed by CD14+ magnetic cell sorting (Miltenyi Biotech). Macrophages were cultivated in X-VIVO 10 Serum-free medium (Cambrex) at a concentration of 1 × 10^6^ cells/ml. The cells were stimulated with 10 ng/ml IL-4 and 100 ng/ml IFN-γ (both from Peprotech) as indicated. The detailed protocol for monocyte isolation is available online (http://www.methods.info/).

Human colorectal cancer cell line WIDR was kindly provided by research group of Prof. Heike Allgayer (Medical Faculty Mannheim, University of Heidelberg) and cultured in McCoys medium (Gibco) supplemented with 10% FBS (Biochrom). RAW 264.7 cells (ATCC CRL-6323) were obtained from American Type Culture Collection and grown in Dulbecco’s modified Eagle’s medium (DMEM) containing 10% FCS, 1% non-essential amino acids, 1% penicillin/streptomycin and 1% sodium pyruvate.

For the generation of RAW 264.7 cell lines stably expressing FoxQ1, the DNA fragment encoding murine FoxQ1 was amplified using High Fidelity *PCR* (Roche) and plasmid pCMV-SPORT6-mouse FoxQ1 (ImaGenes) as a template and primers *mFoxQ1*-*NotI*-fw 5′3′ and *mFoxQ1*-*BamHI*-rv: 5′3′. Further, mFoxQ1 fragment was subcloned into the expression vector pEF6/V5-His Topo (Invitrogen) using NotI and BamHI restriction sites followed by sequencing of the final product. The pEF6/V5-His mFoxQ1 expression construct was transfected in RAW264.7 cells using Nucleofector Kit V and Nucleofector Device (Lonza). Single-cell derived stable clones were selected using blasticidin S (Invitrogen) at the concentration of 6 µg/ml. Stable expression of FoxQ1 was detected by immunofluorescent analysis using anti-V5 ab (Invitrogen). As a negative control, RAW 264.7 were stably transfected with pEF6/V5-His Topo vector.

### RNA isolation and cDNA synthesis

Isolation of total RNA from cells was performed using E.Z.N.A. total RNA Kit I (Omega bio-tek) according to manufacturer’s recommendation. For cDNA synthesis RevertAid H Minus First Strand Synthesis Kit (Fermentas) was used. Prior to cDNA synthesis 0.5 µg RNA were treated with 2 U DNAse I (Fermentas).

### Microarray analysis

For oligonucleotide microarray analysis, total RNA was isolated out of five FoxQ1 and four empty vector RAW 264.7 clones and hybridized on GeneChip Mouse Genome 430 2.0 Array (Affymetrix). The data were submitted to GEO database, accession number of the study is GSE79521. cDNA and cRNA synthesis and hybridization to arrays were performed according to the recommendations of the manufacturer. A Custom CDF Version 12 with Entrez based gene definitions was used to annotate the arrays. The raw fluorescence intensity values were normalized applying quantile normalization. Differential gene expression was analysed based on One-Way ANOVA, using a commercial software package SAS JMP7 Genomics, version 4, from SAS (SAS Institute, Cary, NC, USA). A false positive rate of a = 0.05 with FDR correction was taken as the level of significance.

### RT-qPCR analysis

RT-qPCR analysis was performed to identify relative quantification of gene expression. SybrGreen-based identification of relative gene expression of mouse *Cldn11, Lsp1, and Plxnc1* was performed using SensiMixTM SYBR Low-ROX One-Step Kit (company). The expression of human *FOXQ1*, mouse *Foxq*1, human *CLDN11*, *LSP1*, and *PLXNC1* was assessed using Taqman dual-labeled probes and TaqMan Gene Expression Master Mix (Applied Biosystems). Mouse *Ccr2* was amplified using predesigned TaqMan CCR2 assay (Life technologies). Normalization of gene expression levels was performed using housekeeping genes *GAPDH* or *Actb*. All oligonucleotides used in the present study were from MWG-Biotech and are listed in Supplementary Table 3.

### Immunofluorescence and confocal microscopy

Cells were fixed as described previously^50^. RAW 264.7 cells were stained using anti-V5 antibodies (Invitrogen, cat R960-25) followed by Cy3-conjugated donkey anti-mouse IgG (Dianova). Primary human macrophages and WIDR colorectal cancer cells were stained using goat anti-human FoxQ1 antibody (clone C-16, Santa Cruz Biotechnology) followed by Cy3-conjugated donkey anti-goat IgG (Dianova). Nuclei were visualized using DRAQ5 (Cell Signaling Technology). Leica TCS SP2 and Leica TCS SP8 laser-scanning spectral confocal microscopes equipped with a 63x objective were used for confocal laser-scanning microscopy. Data were acquired and analyzed with Leica Confocal software. All multicolor images were acquired and assembled using a sequential scan mode.

### Phagocytosis and endocytosis assays

For the quantification of the uptake of phagocytic and endocytic ligands, RAW-FoxQ1 cells and RAW-vector cells were plated in 12-well plates in concentration 5 × 10^5^ cells per well. After 18 h, 1 µm size green fluorescent latex beads (Polysciences, Inc.) were added at 50 particles per cell. Incubation with beads was performed at 37 °C for 3 h. For endocytosis acLDL-Alexa488 (Molecular Probes) has been added to cells at the concentration of 5 µg/ml and incubated at 37 °C for 30 min. All incubations were performed in triplicates within one experiment, and 3 independent experiments have been conducted. Uptake of fluorescently-labeled ligands was quantified by flow cytometry using FACS CantoII.

### LPS-induced TNFα production assay

For TNFα secretion assay two RAW FoxQ1 clones with the highest level of FoxQ1 expression and two RAW-vector clones were plated in a 24-well plate (5 × 10^5^ cells per well for 24 h and 1 × 10^6^ cells per well for 3 and 6 h time point) and stimulated with 1 µg/ml LPS. TNFα level in supernatants was determined by mouse TNFα DuoSet ELISA Kit (R&D systems) according to manufacturer’s recommendation.

### Cell migration assay

A forty-eight-well microchemotaxis chamber (Neuroprobe) and a polycarbonate filter with 8 µm pore size were used. The cells were added to the chamber (3 × 10^4^ cells/well) and allowed to migrate towards MCP-1 (R&D Systems) at the concentration of 100 ng/ml. After 2 h incubation at 37 °C and 5% CO_2_, the cells that had not migrated and remained on the upper side of membrane were removed by scraping. The cells that migrated and were located on the down-side of membrane were fixed with 4% paraformaldehyde (Fluka). Fixed membranes were stained with cell stain solution (Cell Biolabs, Inc.). Migrated cells were counted in 3 randomly selected fields per well under the microscope using 10x magnification. Five RAW-mFoxQ1 clones and five RAW-empty vector clones were compared in triplicates (three wells per clone). The experiment was repeated three times.

### Statistical analysis

The difference between experimental groups was analyzed using two-tailed Student’s t-test, Mann- Whitney U test, ANOVA with Tukey’s and Sidak’s post-hoc tests, or Kruskal-Wallis test with Dunn’s post-hoc test as indicated in the text and figure legends. The difference was considered statistically significant at p < 0.05 level.

### Data availability

The datasets generated during and/or analyzed during the current study are available from the corresponding author on reasonable request.

## Electronic supplementary material

Supplementary data

## References

[1] Huang R (2016). Increase of infiltrating monocytes in the livers of patients with chronic liver diseases. Discovery medicine.

[2] Kiekens RC (2001). Heterogeneity within tissue-specific macrophage and dendritic cell populations during cutaneous inflammation in atopic dermatitis. The British journal of dermatology.

[3] Shi C, Pamer EG (2011). Monocyte recruitment during infection and inflammation. Nature reviews. Immunology.

[4] Deshmane SL, Kremlev S, Amini S, Sawaya BE (2009). Monocyte chemoattractant protein-1 (MCP-1): an overview. Journal of interferon & cytokine research: the official journal of the International Society for Interferon and Cytokine Research.

[5] Martinez FO, Gordon S (2014). The M1 and M2 paradigm of macrophage activation: time for reassessment. F1000prime reports.

[6] Gratchev A, Kzhyshkowska J, Utikal J, Goerdt S (2005). Interleukin-4 and dexamethasone counterregulate extracellular matrix remodelling and phagocytosis in type-2 macrophages. Scandinavian journal of immunology.

[7] Guttman-Yassky E, Dhingra N, Leung DY (2013). New era of biologic therapeutics in atopic dermatitis. Expert opinion on biological therapy.

[8] Maes T, Joos GF, Brusselle GG (2012). Targeting interleukin-4 in asthma: lost in translation?. American journal of respiratory cell and molecular biology.

[9] Renz H (1992). Enhanced IL-4 production and IL-4 receptor expression in atopic dermatitis and their modulation by interferon-gamma. The Journal of investigative dermatology.

[10] Nomura T, Kabashima K (2016). Advances in atopic dermatitis in 2015. J Allergy Clin Immunol.

[11] Barton, M. & Sidbury, R. Advances in understanding and managing atopic dermatitis. *F1000Research* **4**, 10.12688/f1000research.6972.1 (2015). 10.12688/f1000research.6972.1 PMC4753999 26918129

[12] Kasraie S, Werfel T (2013). Role of macrophages in the pathogenesis of atopic dermatitis. Mediators of inflammation.

[13] Leung DY, Boguniewicz M, Howell MD, Nomura I, Hamid QA (2004). New insights into atopic dermatitis. The Journal of clinical investigation.

[14] Xue J (2014). Transcriptome-based network analysis reveals a spectrum model of human macrophage activation. Immunity.

[15] Abba M (2013). Unraveling the role of FOXQ1 in colorectal cancer metastasis. Molecular cancer research: MCR.

[16] Feng J (2014). Involvement of FoxQ1 in NSCLC through regulating EMT and increasing chemosensitivity. Oncotarget.

[17] Qiao Y (2011). FOXQ1 regulates epithelial-mesenchymal transition in human cancers. Cancer research.

[18] Zhang H (2011). Forkhead transcription factor foxq1 promotes epithelial-mesenchymal transition and breast cancer metastasis. Cancer research.

[19] Gratchev A (2006). Mphi1 and Mphi2 can be re-polarized by Th2 or Th1 cytokines, respectively, and respond to exogenous danger signals. Immunobiology.

[20] Sehra S (2008). IL-4 is a critical determinant in the generation of allergic inflammation initiated by a constitutively active Stat6. Journal of immunology.

[21] Shi VY, Bao L, Chan LS (2012). Inflammation-driven dermal lymphangiogenesis in atopic dermatitis is associated with CD11b+ macrophage recruitment and VEGF-C up-regulation in the IL-4-transgenic mouse model. Microcirculation.

[22] Kzhyshkowska J, Neyen C, Gordon S (2012). Role of macrophage scavenger receptors in atherosclerosis. Immunobiology.

[23] Agarwal R (2009). Silencing of claudin-11 is associated with increased invasiveness of gastric cancer cells. PloS one.

[24] Chabbert-de Ponnat I (2005). Soluble CD100 functions on human monocytes and immature dendritic cells require plexin C1 and plexin B1, respectively. International immunology.

[25] Hwang SH (2015). Leukocyte-specific protein 1 regulates T-cell migration in rheumatoid arthritis. Proceedings of the National Academy of Sciences of the United States of America.

[26] Liu L (2005). LSP1 is an endothelial gatekeeper of leukocyte transendothelial migration. The Journal of experimental medicine.

[27] Stout RD, Suttles J (2004). Functional plasticity of macrophages: reversible adaptation to changing microenvironments. Journal of leukocyte biology.

[28] Stout RD, Watkins SK, Suttles J (2009). Functional plasticity of macrophages: *in situ* reprogramming of tumor-associated macrophages. Journal of leukocyte biology.

[29] Schultze JL (2016). Reprogramming of macrophages–new opportunities for therapeutic targeting. Current opinion in pharmacology.

[30] Davis MJ (2013). Macrophage M1/M2 polarization dynamically adapts to changes in cytokine microenvironments in Cryptococcus neoformans infection. mBio.

[31] Kzhyshkowska J, Yin S, Liu T, Riabov V, Mitrofanova I (2016). Role of chitinase-like proteins in cancer. Biological chemistry.

[32] Roszer T (2015). Understanding the Mysterious M2 Macrophage through Activation Markers and Effector Mechanisms. Mediators of inflammation.

[33] Gratchev A (2013). Novel monocyte biomarkers of atherogenic conditions. Current pharmaceutical design.

[34] Kaburagi Y (2001). Enhanced production of CC-chemokines (RANTES, MCP-1, MIP-1alpha, MIP-1beta, and eotaxin) in patients with atopic dermatitis. Archives of dermatological research.

[35] Vogel DY (2014). Macrophages migrate in an activation-dependent manner to chemokines involved in neuroinflammation. Journal of neuroinflammation.

[36] Belloni B, Andres C, Ollert M, Ring J, Mempel M (2008). Novel immunological approaches in the treatment of atopic eczema. Current opinion in allergy and clinical immunology.

[37] Sumimoto S, Kawai M, Kasajima Y, Hamamoto T (1992). Increased plasma tumour necrosis factor-alpha concentration in atopic dermatitis. Archives of disease in childhood.

[38] Walling HW, Swick BL (2010). Update on the management of chronic eczema: new approaches and emerging treatment options. Clinical, cosmetic and investigational dermatology.

[39] Lampinen M (2001). IL-5 and TNF-alpha participate in recruitment of eosinophils to intestinal mucosa in ulcerative colitis. Dig Dis Sci.

[40] Lukacs NW, Strieter RM, Chensue SW, Widmer M, Kunkel SL (1995). TNF-alpha mediates recruitment of neutrophils and eosinophils during airway inflammation. Journal of immunology.

[41] Rossol M (2013). Tumor necrosis factor receptor type I expression of CD4+ T cells in rheumatoid arthritis enables them to follow tumor necrosis factor gradients into the rheumatoid synovium. Arthritis Rheum.

[42] Watanabe C (2002). Spatial heterogeneity of TNF-alpha-induced T cell migration to colonic mucosa is mediated by MAdCAM-1 and VCAM-1. Am J Physiol Gastrointest Liver Physiol.

[43] Zhang K, Gharaee-Kermani M, McGarry B, Remick D, Phan SH (1997). TNF-alpha-mediated lung cytokine networking and eosinophil recruitment in pulmonary fibrosis. Journal of immunology.

[44] Mackay F, Loetscher H, Stueber D, Gehr G, Lesslauer W (1993). Tumor necrosis factor alpha (TNF-alpha)-induced cell adhesion to human endothelial cells is under dominant control of one TNF receptor type, TNF-R55. The Journal of experimental medicine.

[45] Shulman Z (2011). Transendothelial migration of lymphocytes mediated by intraendothelial vesicle stores rather than by extracellular chemokine depots. Nat Immunol.

[46] Teo GS (2012). Mesenchymal stem cells transmigrate between and directly through tumor necrosis factor-alpha-activated endothelial cells via both leukocyte-like and novel mechanisms. Stem Cells.

[47] Woodfin A (2009). Endothelial cell activation leads to neutrophil transmigration as supported by the sequential roles of ICAM-2, JAM-A, and PECAM-1. Blood.

[48] Xia L (2014). Forkhead box Q1 promotes hepatocellular carcinoma metastasis by transactivating ZEB2 and VersicanV1 expression. Hepatology.

[49] Gratchev A (2004). The receptor for interleukin-17E is induced by Th2 cytokines in antigen-presenting cells. Scandinavian journal of immunology.

[50] Kzhyshkowska J (2004). Stabilin-1 localizes to endosomes and the trans-Golgi network in human macrophages and interacts with GGA adaptors. Journal of leukocyte biology.

