# Supplementary material for: IL-4 driven transcription factor FoxQ1 is expressed by monocytes in atopic dermatitis and stimulates monocyte migration
Source: Sci Rep. 2017 Dec 4;7:16847. doi: 10.1038/s41598-017-17307-z (PMC5715145; doi:10.1038/s41598-017-17307-z)
Supplement: Supplementary file 1 — Supplementary data [file 41598_2017_17307_MOESM1_ESM.pdf]

Manuscript title: IL-4 driven transcription factor FoxQ1 is expressed by monocytes in atopic dermatitis and stimulates monocyte migration

Authors: Ilja Ovsiy, Vladimir Riabov, Ioannis Manousaridis, Julia Michel, Kondaiah Moganti, Shuiping Yin, Tengfei Liu, Carsten Sticht, Elisabeth Kremmer, Martin C. Harmsen, Sergij Goerdt, Alexei Gratchev, and Julia Kzhyshkowska

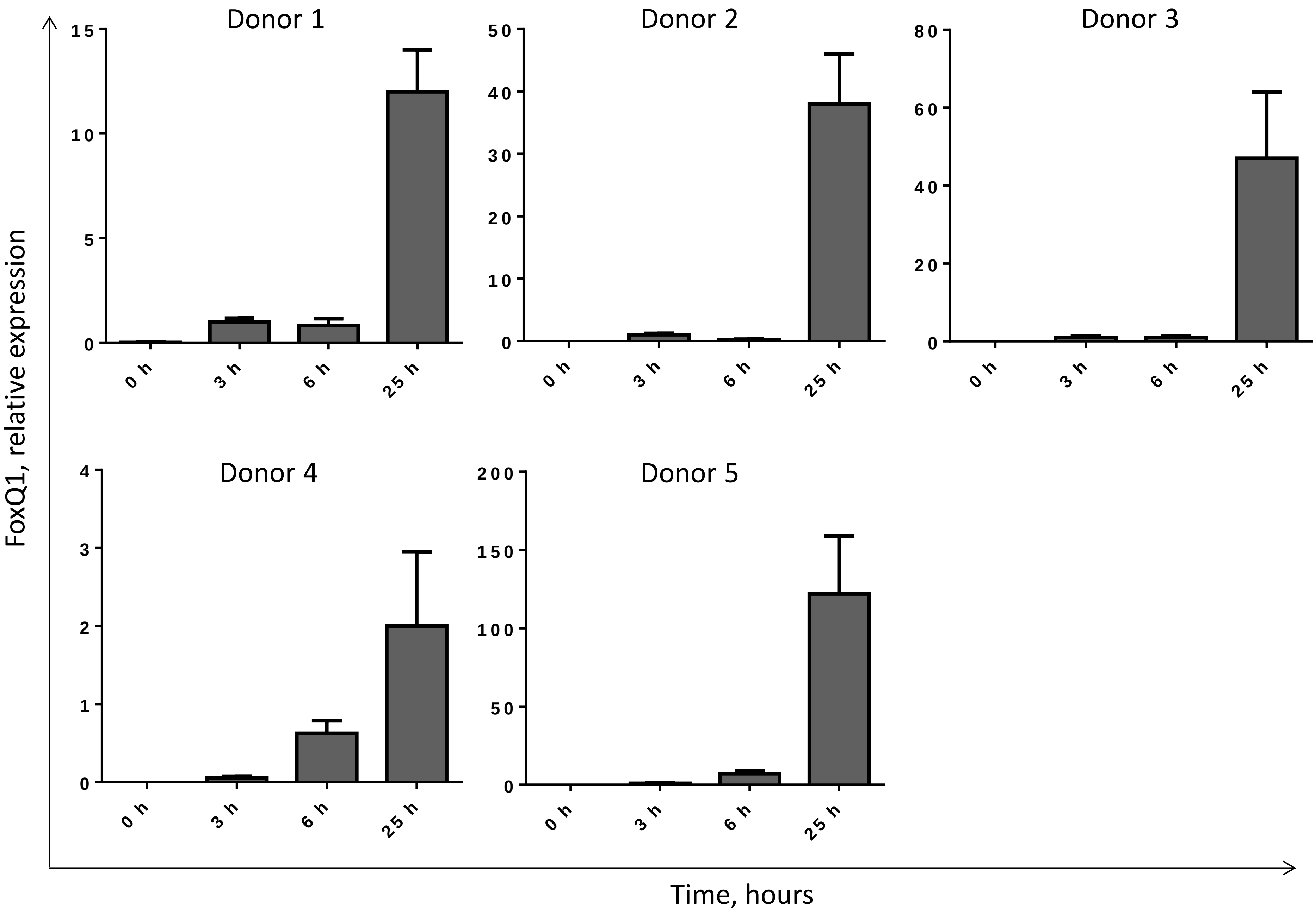

Supplementary Figure 1. The dynamics of FoxQ1 expression in human monocytes after IL4 stimulation. The monocytes of 5 healthy donors were stimulated with IL4 (10 ng/ml) and analyzed using qRT-PCR after 0h, 3h, 6h, and 25h after stimulation. The data are presented as mean±SD of triplicates for individual donors.

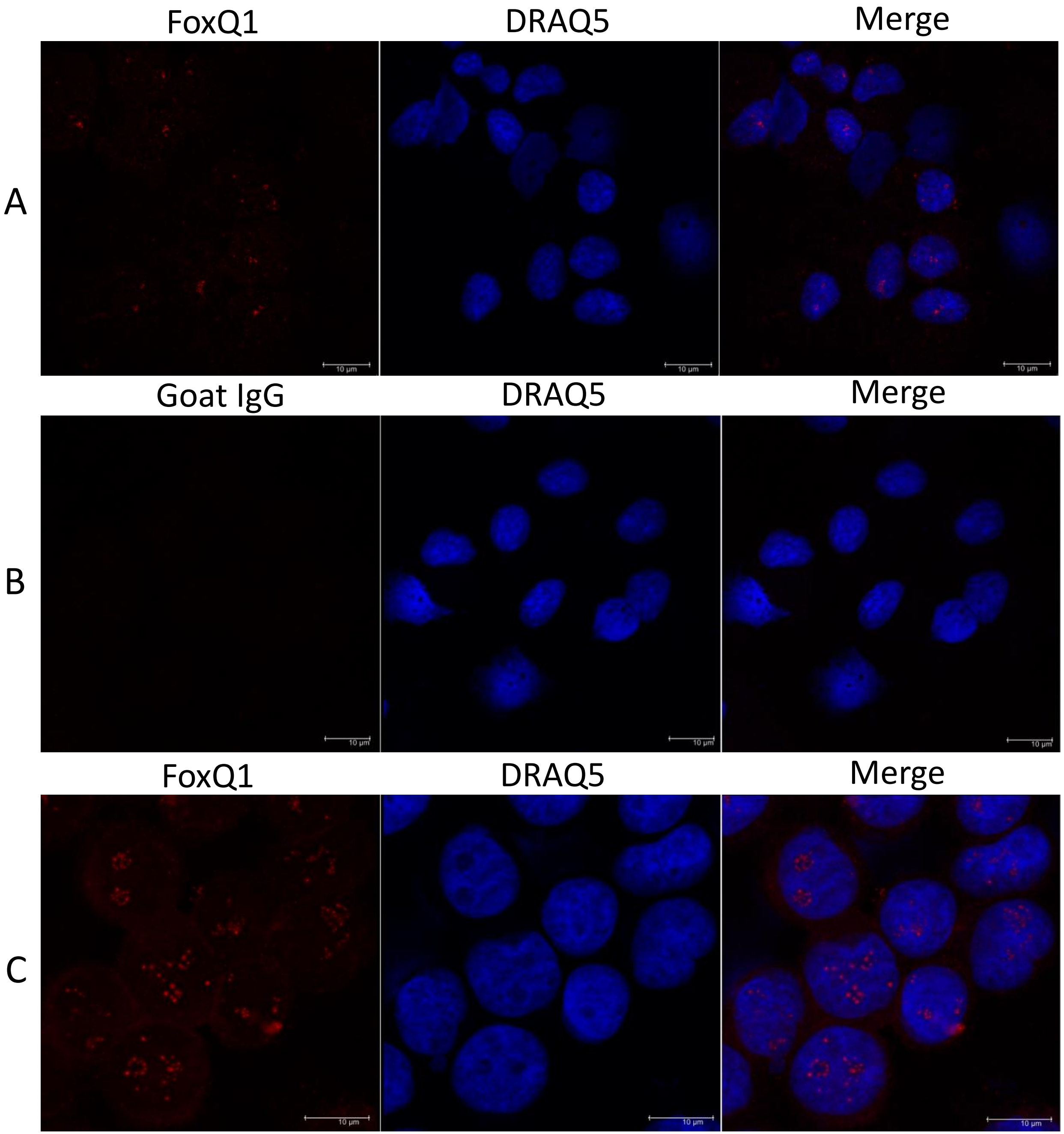

Supplementary Figure 2. Expression of FoxQ1 protein in human macrophages and WIDR cancer cells. Primary human monocyte-derived macrophages were stimulated with IL4 for 6 days and stained using goat anti-FoxQ1 abs (A) or goat IgG isotype control (B). WIDR human colorectal cancer cell line was stained using FoxQ1 abs and served as a positive control (C). Nuclei were visualized using DRAQ5. Scale bars: 10

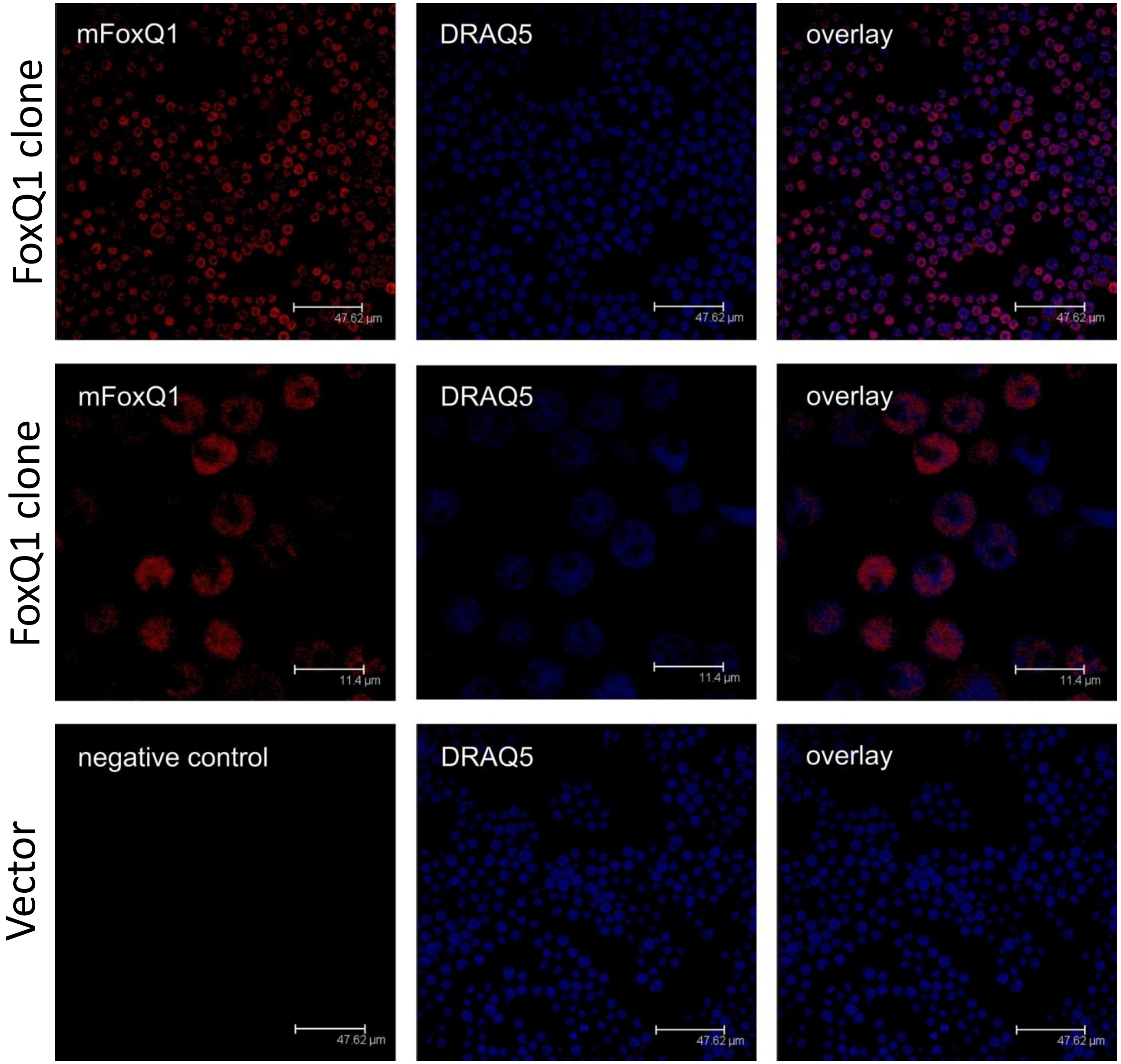

Supplementary Figure 3. FoxQ1 expression in RAW 264.7 cells stably transfected with mouse FoxQ1 expression construct. Cells were fixed with PFA and analyzed by IF/confocal microscopy (red: anti-V5/anti-mouse Cy3; blue: DRAQ5). Vector-transfected cells were used as a negative control

Supplementary Table 1. The list of differentially expressed (upregulated) genes responsible for cytoskeletal dynamics in RAW264.7-FoxQ1 clones.

Accession number	Gene Symbol	Gene Name	Diff. of Exp.	-log10 (p-value)
NM_001166406	Kif20a	kinesin family member 20A	2,82	11,68
NM_024245	Kif23	kinesin family member 23	2,07	9,11
NM_145588	Kif22	kinesin family member 22	2,04	9,17
NM_008446	Kif4	kinesin family member 4	1,71	9,46
NM_010615	Kif11	kinesin family member 11	1,65	8,37
NM_197959	Kif18b	kinesin family member 18B	1,60	6,99
NM_134471	Kif2c	kinesin family member 2C	1,43	9,76
NM_183046	Kif20b	kinesin family member 20B	1,30	6,85
NM_139303	Kif18a	kinesin family member 18A	1,06	6,14
NM_028390	Anln	anillin, actin binding protein	1,50	8,89
NM_133755	Tubgcp2	tubulin, gamma complex associated protein	1,62	10,03
NM_134024	Tubg1	tubulin, gamma 1	1,10	6,57
NM_011655	Tubb5	tubulin, beta 5	0,98	6,07
NM_019756	Tubd1	tubulin, delta 1	0,67	6,48
NM_009446	Tuba3a	tubulin, alpha 3A	0,66	5,89
NM_001033484	Iqgap3	IQ motif containing GTPase activating protein	1,69	5,81
NM_001004140	Ckap2	cytoskeleton associated protein 2	1,28	9,28
NM_001165989	Ckap5	cytoskeleton associated protein 5	1,14	6,25
NM_181589	Ckap2l	cytoskeleton associated protein 2-like	1,11	8,94
NM_019670	Diap3	diaphanous homolog 3 (Drosophila)	0,79	6,93
NM_023142	Arpc1b	actin related protein 2/3 complex	0,68	7,13

Supplementary Table 2. The list of differentially expressed (downregulated) genes responsible for migration and metastasis in RAW264.7-FoxQ1 clones.

**Accession number**	**Gene Symbol**	**Gene Name**	**Diff. of Exp.**	**-log10 (p-value)**
NM_198862	Nlgn2	neuroligin 2	-0,52	7,29
NM_008397	Itga6	integrin alpha 6	-1,35	7,90
NM_010576	Itga4	integrin alpha 4	-1,69	6,34
NM_008206	H2-Oa	histocompatibility 2, O region alpha	-0,90	5,95
NM_001167745	Wasl	Wiskott-Aldrich syndrome-like (human)	-0,55	6,65
NM_009148	Exoc4	exocyst complex component 4	-0,52	6,32
NM_001161746	Tnfrsf12a	tumor necrosis factor receptor superfamily	-0,68	5,59
NM_009427	Tob1	transducer of ErbB-2.1	-0,40	5,69
NM_001136071	Lsp1	lymphocyte specific 1	-2,06	6,47
NM_177725	Lrrc8a	leucine rich repeat containing 8A	-0,42	6,74
NM_001136055	Cd82	CD82 antigen	-0,82	8,34
NM_021028	Tk2	thymidine kinase 2, mitochondrial	-0,65	5,62
NM_018797	Plxnc1	plexin C1	-0,86	7,85
NM_025622	Lgals2	lectin, galactose-binding, soluble 2	-0,49	5,64
NM_008770	Cldn11	claudin 11	-4,28	10,30

Supplementary Table 3. List of oligonucleotides for RT-qPCR analysis

	**Gene name**	**Primer sequence**
F	murine FoxQ1	caaggtgctgcgcgaccc
R	murine FoxQ1	tgtggtccggtggctgagg
Pr	murine FoxQ1	ctactggatgctcaaccccaacagcgaatacaccttcgcc
F	human FoxQ1	gatgcttcactgtgccaaaa
R	human FoxQ1	gcccaaggagaccacagtta
Pr	human FoxQ1	tgcaggcaacgggctacagc
F	murine claudin 11	atcctcatccttccaggctacg
R	murine claudin 11	gagaactgtcaacagcagcaag
F	murine PLXNC1	caagcgactgagcacaac
R	murine PLXNC1	cggtccccaagaacagaac
F	murine LSP1	tcaaagcgagagaccagaggagaaac
R	murine LSP1	caacggtgtcttctaaggccaaagg
F	human LSP1	agacatgagcaagaaaagcctctg
R	human LSP1	ccttccacaagcaccttctcatac
Pr	human LSP1	tgctcttaattgttgatgaggtcttggagcctcccttc
F	human PLXNC1	tgtcgagaagacatcaggag
R	human PLXNC1	cccgttactatcacgttgc
Pr	human PLXNC1	ccaaggagaacaaggggaacagaaccaa
F	murine GAPDH	catccatgacaactttggcattgt
R	murine GAPDH	cagtcttctgggtggcagtga
Pr	murine GAPDH	aaggactcatgaccacagtccatgcc
F	human GAPDH	tccatgacaactttggtatcgt
R	human GAPDH	cagtcttctgggtggcagtga
Pr	human GAPDH	aaggactcatgaccacagtccatgcc
F	murine beta actin	tgctctccctcacgccatcc
R	murine beta actin	cggaaccgctcgttgccaatag

Pr – Taqman Probe
